# Nanomaterial-Based Synaptic Optoelectronic Devices for In-Sensor Preprocessing of Image Data

**DOI:** 10.1021/acsomega.3c00440

**Published:** 2023-02-03

**Authors:** Minkyung Lee, Hyojin Seung, Jong Ik Kwon, Moon Kee Choi, Dae-Hyeong Kim, Changsoon Choi

**Affiliations:** † Center for Optoelectronic Materials and Devices, Post-silicon Semiconductor Institute, Korea Institute of Science and Technology (KIST), Seoul 02792, Republic of Korea; ‡ Center for Nanoparticle Research, Institute for Basic Science (IBS), Seoul 08826, Republic of Korea; § School of Chemical and Biological Engineering, Institute of Chemical Processes, Seoul National University, Seoul 08826, Republic of Korea; ∥ School of Materials Science and Engineering, Ulsan National Institute of Science and Technology (UNIST), Ulsan 44919, Republic of Korea; ⊥ Department of Materials Science and Engineering, Seoul National University, Seoul 08826, Republic of Korea

## Abstract

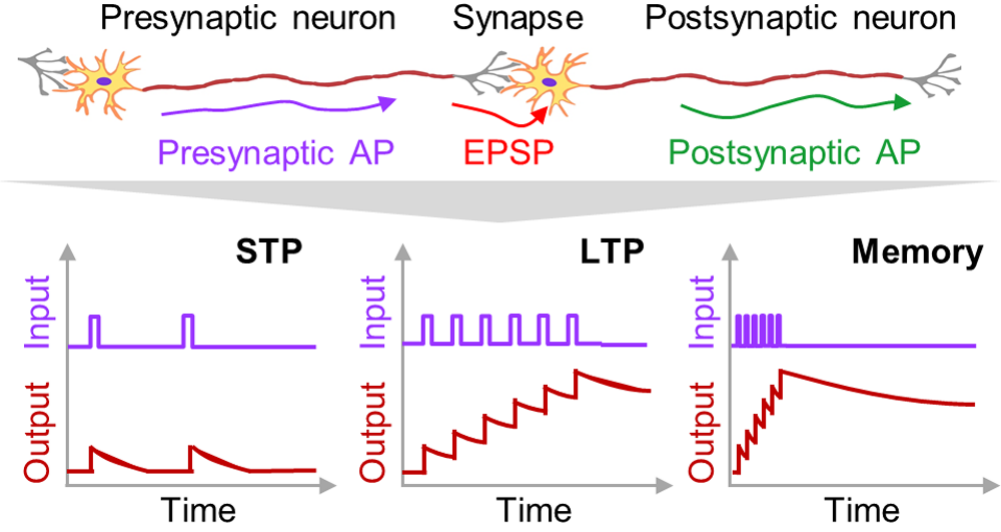

With the advance in information technologies involving machine vision applications, the demand for energy- and time-efficient acquisition, transfer, and processing of a large amount of image data has rapidly increased. However, current architectures of the machine vision system have inherent limitations in terms of power consumption and data latency owing to the physical isolation of image sensors and processors. Meanwhile, synaptic optoelectronic devices that exhibit photoresponse similar to the behaviors of the human synapse enable in-sensor preprocessing, which makes the front-end part of the image recognition process more efficient. Herein, we review recent progress in the development of synaptic optoelectronic devices using functional nanomaterials and their unique interfacial characteristics. First, we provide an overview of representative functional nanomaterials and device configurations for the synaptic optoelectronic devices. Then, we discuss the underlying physics of each nanomaterial in the synaptic optoelectronic device and explain related device characteristics that allow for the in-sensor preprocessing. We also discuss advantages achieved by the application of the synaptic optoelectronic devices to image preprocessing, such as contrast enhancement and image filtering. Finally, we conclude this review and present a short prospect.

## 1 Introduction

Recent advances in image acquisition^1−3^ and data processing^4,5^ technologies have enabled diverse machine vision applications including facile object detection^6−8^ and accurate image recognition,^9,10^ leading to a new era in the development of surveillance, self-driving vehicles, and autonomous robotics technologies.^11,12^ Simultaneously, the amount of image data to be acquired and processed by the image sensing and processing device for realizing the machine vision application has been exponentially growing, significantly increasing the computational burden.^6,13^ However, the conventional architecture of the machine vision system, where the front-end image sensor and the back-end processor are physically separated, involves inefficiency in terms of power consumption and data latency.^14^ This is because the considerable amount of image data in the entire time domain should be acquired by the image sensor and then transferred to the processor for image data processing and recognition, consequently consuming significant energy and processing time.^15,16^

One promising solution for this inefficiency that originated from the system configuration is to adopt a novel image-sensing and data-processing platform that emulates the human vision and image recognition system.^4,17,18^ The human retina performs first-stage image preprocessing by the bipolar and ganglion cells as well as performs image acquisition by the photoreceptor cells.^19,20^ Inspired by this image acquisition and preprocessing mechanism of the retina, researchers have made significant efforts to add the image data preprocessing functions (e.g., contrast enhancement^7,10^ and image filtering^5,14^) into the image sensors, intending to integrate the front-end image preprocessing function into the image sensing device.^21^ Such image data preprocessing in the image sensor is called in-sensor preprocessing,^22,23^ where the image data can be preprocessed simultaneously during the image-sensing step without additional computations, thus reducing the computational burden in the image recognition step.^6,24^

Synaptic optoelectronic devices, whose photoresponses are similar to the behaviors of the human synapse (e.g., short-term plasticity and long-term potentiation),^25,26^ can achieve the image acquisition and in-sensor preprocessing through a single readout operation.^15^ The computations required in the conventional machine vision systems to perform the image preprocessing can be reduced by the in-sensor preprocessing, thereby enhancing the overall efficiency of the machine vision operation.^22^ However, such synaptic optoelectronic properties cannot be achieved by using conventional semiconducting materials (e.g., Si and III–IV semiconductors).^2,3^ Therefore, novel optoelectronic materials and device structures have been intensively researched to confer synapse-inspired properties on the image-sensing device.^27^

Various types of functional nanomaterials, such as amorphous oxide semiconductors (AOSs),^28,29^ two-dimensional (2D) materials,^9^ semiconducting nanoparticles,^10^ and halide perovskites,^30^ have been studied for the development of photodetectors with synaptic photoresponses. Thus, it has been found that the intrinsic characteristics of functional nanomaterials and their interfacial properties (e.g., oxygen vacancy ionization,^31^ interfacial charge trapping,^32,33^ and heterojunction charge transfer^30^) enable such unconventional photoresponses (e.g., time-dependent photocurrent generation^21^ and persistent photocurrent (PPC)^13^). Therefore, optoelectronic devices featuring synapse-inspired properties (e.g., photon-triggered synaptic plasticity and memory effects) and providing in-sensor preprocessing functions could be developed.^34^

Here, we review recent progress in the synaptic optoelectronic devices, with a particular focus on the unique roles of nanomaterials and their interfacial characteristics for the synaptic photoresponses. Among various types of nanomaterials, we review representative material groups, such as AOSs, 2D materials, semiconducting nanoparticles, and halide perovskites. The intrinsic characteristics originating from the materials or their interfaces with adjacent device layers confer synapse-inspired properties to the photodetecting devices. We explain the detailed characteristics of such nanomaterials along with their fundamental physics that induce unconventional photoresponses, and describe the properties of synaptic optoelectronic devices triggered by optical stimuli. We then summarize advantageous results by the application of the synaptic optoelectronic devices to the in-sensor preprocessing of the acquired image data, such as contrast enhancement and image filtering, which are key requirements of high-performance machine vision. Finally, we include a short conclusion section discussing the future prospects.

## 2 Nanomaterial-Based Synaptic Optoelectronic Devices

Newly emerged synaptic optoelectronic devices have favorable features in achieving machine vision applications efficiently.^24^ This is due to their synaptic properties that allow in-sensor preprocessing during acquisition of image data, which can reduce the computational burden. However, synaptic optoelectronic devices cannot be realized by using the conventional semiconducting materials used in traditional CMOS technology (e.g., silicon).^35^ These conventional optoelectronic devices typically show rapid photoresponse and instant photocurrent decay,^36,37^ which are different from the time-dependent and persistent behavior of human synapses.^38^ In this regard, research efforts are demanded in terms of material selection and interfacial engineering for the development of synaptic optoelectronic devices.

**Figure 1:**
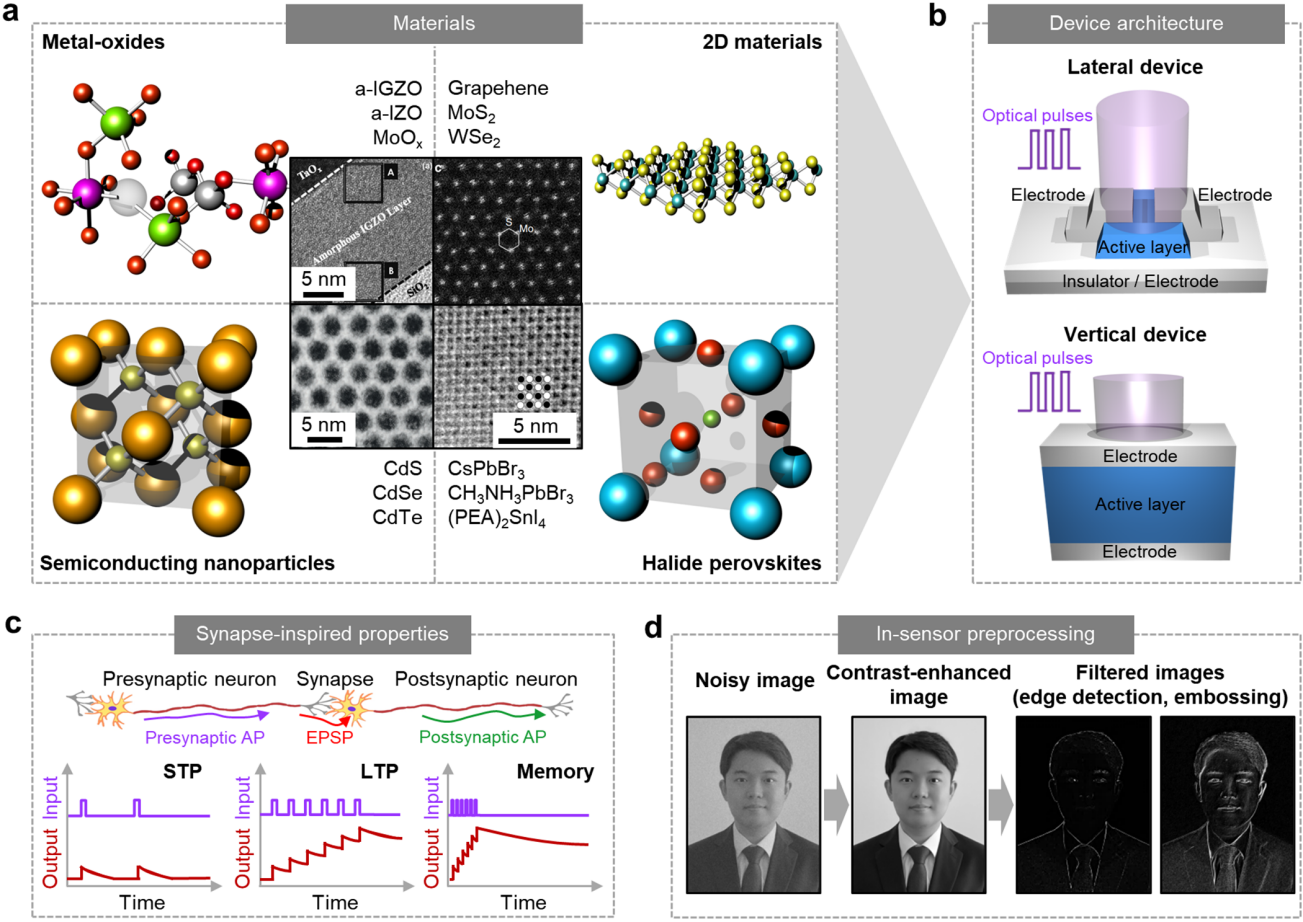
Synaptic optoelectronic devices for in-sensor preprocessing. (a) Schematic of the crystal structure of functional nanomaterials (e.g., AOSs, 2D materials, semiconducting nanoparticles, and halides perovskites) used in the synaptic optoelectronic devices. Inset shows the transmission electron microscopy images of such nanomaterials. Reproduced with permission from refs (39−42). Copyright 2014, 2015, 2017 Nature Publishing Group (NPG) and 2020 American Association for the Advanced Science (AAAS). (b) Schematic of the architectures (e.g., lateral and vertical device design) of synaptic optoelectronic devices. (c) Representative synapse-inspired properties (e.g., STP, LTP, and memory effect) of synaptic optoelectronic devices induced by the optical pulses with different frequency. (d) In-sensor preprocessing performed using the synaptic optoelectronic devices to obtain a contrast-enhanced image and filter the acquired images.

Various types of functional nanoscale materials have been extensively investigated due to their inherent material and interfacial characteristics that can allow synaptic photoresponses as well as high-performance optical and electrical properties (Figure 1a).^39−42^ Such nanoscale materials include AOS thin films (e.g., amorphous indium gallium zinc oxide (a-IGZO), amorphous indium zinc oxide (a-IZO), and MoO_*x*_),^7,13,17^ 2D materials (e.g., graphene, MoS_2_, and WSe_2_),^43,44^ semiconducting nanoparticles (e.g., CdSe, CdS, and CdTe),^10,45^ and halide perovskites (e.g., CsPbBr_3_, CH_3_NH_3_PbBr_3_, and (PEA)_2_SnI_4_).^46,47^ These nanomaterials exhibit unique physical phenomena, such as ionization/deionization of oxygen vacancies (AOSs), interfacial charge trapping/detrapping (2D materials), and charge transfer at the heterointerface (semiconducting nanoparticles and halide perovskites). Such processes occur gradually over a long period of time because of their high activation energy. Therefore, the photocurrent generation is not rapid, which is similar to the time-dependent behavior of the synapse. The photocurrent relaxation, which brings the materials or interfaces to their initial state, also occurs slowly since it is a thermally activated process that needs high activation energy, thus allowing the PPC behavior.^13^

The synaptic optoelectronic devices can adopt diverse device types with lateral or vertical architectures, such as phototransistors,^17^ photodiodes,^8^ and optoelectronic memory^7^ (Figure 1b). In such devices, the functional nanomaterials have been used as an active channel, of which conductance is modulated by the light irradiation and maintained for a long period of time. These devices exhibit synapse-like photoresponse (e.g., short-term plasticity (STP), long-term potentiation (LTP), and memory effect) upon the irradiation of optical inputs (Figure 1c).^25^ In the synapse, the neurotransmitter release is induced by the presynaptic actional potentials (APs), and the released neurotransmitters induce the generation of the postsynaptic potential.^48^ The amount of neurotransmitter release is dependent on the synaptic weight, which can be temporally or relatively permanently enhanced by the repetitive presynaptic APs (i.e., STP or LTP).^15^ Similarly, in the synaptic optoelectronic devices, large and long-lasting conductance is induced by optical inputs with high frequency, or a low and temporal conductance is induced by optical inputs with low frequency.

Therefore, synaptic optoelectronic devices perform in-sensor preprocessing, similar to retinal neurons that preprocess acquired visual information before transmitting it to the visual cortex.^49^ Such synaptic optoelectronic devices with photon-triggered synaptic plasticity can reduce background noise and consequently enhance contrast, deriving a preprocessed image from the sequential noisy input images (Figure 1d, middle).^21^ This contrast-enhanced image is helpful for high-accuracy image recognition. In addition, a crossbar array of the synaptic optoelectronic devices can be used to extract the unique features of the image data, such as edge detection and image embossing. For example, the array can conduct analog vector-matrix multiplications for image filtering (Figure 1d, right).^14^

In the following sections, the inherent characteristics of nanomaterials, useful for the development of synaptic optoelectronic devices, will be explained first. Then, we will describe the fundamental physical phenomena and mechanism observed in homogeneous materials and heterogeneous material interfaces that introduce synaptic optoelectronic properties. Finally, the properties of synaptic optoelectronic devices, enabled by the functional nanomaterials, will be discussed.

### 2.1 Amorphous Oxide Semiconductors for Synaptic Optoelectronic Devices

**Figure 2:**
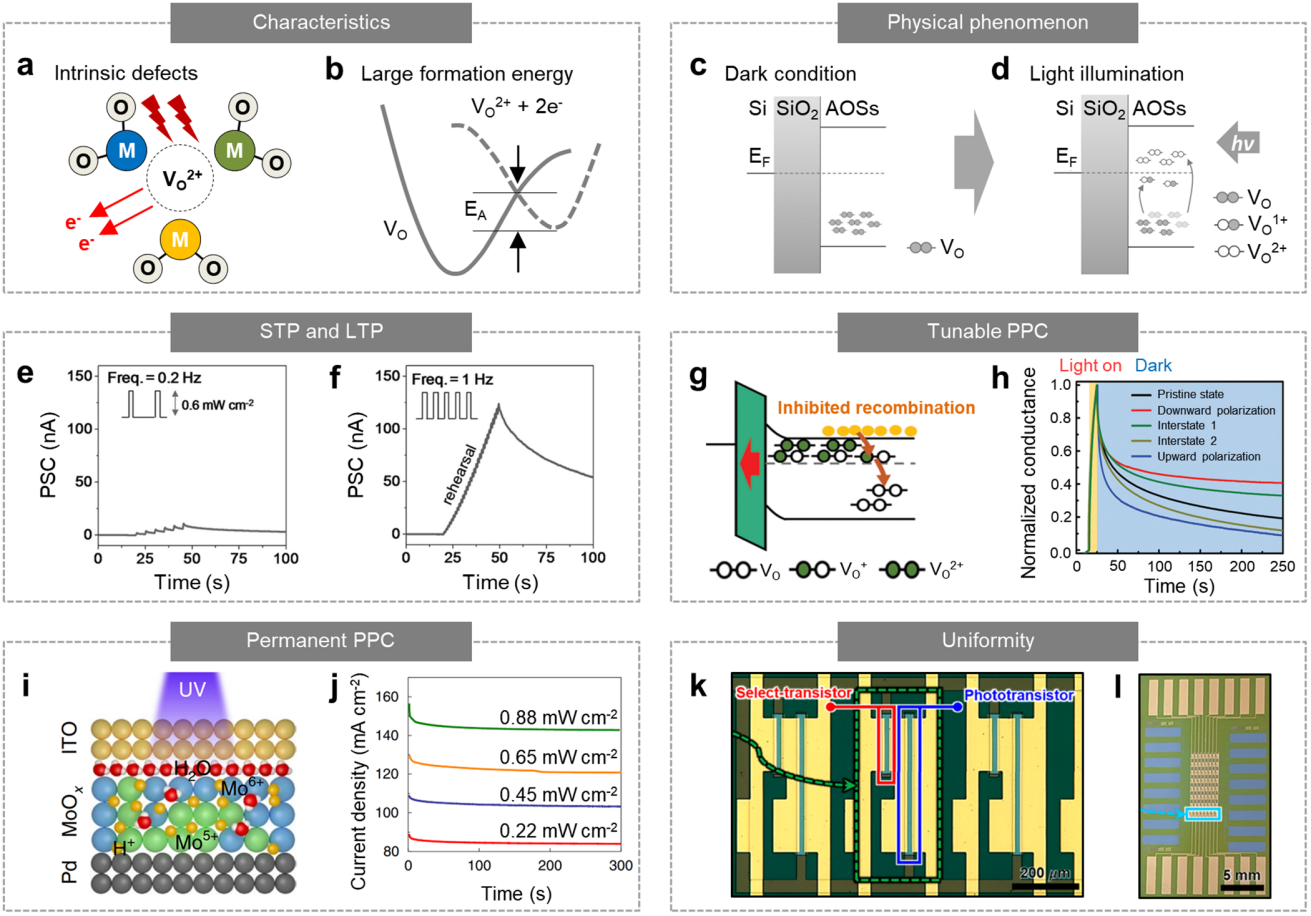
Synaptic optoelectronic devices based on amorphous oxide semiconductors. (a) Schematic of ionization of oxygen vacancies in AOSs by light irradiation. (b) Energy band diagram for the ionization of oxygen vacancies in a-IGZO. (c, d) Energy band diagram of AOSs with deep-level defect states (e.g., oxygen vacancies) under dark (c) and light illumination conditions (d). (e) STP behavior of an a-IGZO phototransistor in response to UV pulses with low frequency (pulse width = 0.5 s, frequency = 0.2 Hz). (f) LTP behavior of an a-IGZO phototransistor in response to the UV pulses with high frequency (pulse width = 0.5 s, frequency = 1 Hz). Reproduced with permission from ref (13). Copyright 2017 Wiley-VCH. (g) Energy band diagram of an a-IGZO phototransistor using HfZrO_*x*_ dielectric. The recombination reaction near the interface between a-IGZO and HfZrO_*x*_ is inhibited because of the electron depletion caused by the upward polarized HfZrO_*x*_. (h) Photocurrent generation and decaying characteristics of an a-IGZO phototransistor with ferroelectric HfZrO_*x*_ dielectric that shows different polarization states. Reproduced with permission from ref (58). Copyright 2020 Wiley-VCH. (i) Schematic of a cross-sectional structure of ORRAM. Mo^6+^ is transformed to Mo^5+^ upon the irradiation of UV light, resulting in the transition of ORRAM from HRS to LRS. (j) Photocurrent decay characteristics of ORRAM depending on incident light intensities. Reproduced with permission from ref (7). Copyright 2019 NPG. (k) Optical microscopy image of the pixels in an active-matrix synaptic optoelectronic device array. Each pixel consists of a select transistor (red box) and a synaptic phototransistor (blue box). (l) Photograph of the active-matrix form of an 8 × 8 synaptic optoelectronic device array. Reproduced with permission from ref (17). Copyright 2021 American Chemical Society (ACS).

AOSs (e.g., a-IGZO and a-IZO) have been widely used in electronic and optoelectronic devices, such as thin-film transistors, because of their high carrier mobility, low processing temperature, and excellent uniformity.^50−52^ In particular, AOSs have also been widely used in synaptic optoelectronic devices because of their inherent defects (e.g., oxygen vacancies (V_O_)) that lead to an unconventional photoresponse for realizing synapse-inspired properties (Figure 2a).^26,53,54^ It is enabled by the defect-related physical phenomenon (e.g., V_O_ ionization and phase transformation) that demands large energy to overcome the activation energy (Figure 2b). For example, a-IGZO has V_O_ in deep-level energy states (Figure 2c). V_O_ in a-IGZO can be ionized, contributing free electrons to the conduction band (V_O_ → V_O_^2+^ + 2e^–^) and thus enhancing the conductivity (Figure 2d).^55^ Such V_O_ ionization can be facilitated by optical irradiation, resulting in photoconductivity. However, the photocurrent generation is dependent on the light dosage because this process is suppressed by the high activation energy (Figure 2d).^56^ In addition, the recombination reaction (V_O_^2+^ + 2e^–^ → V_O_) does not instantly occur after removing the optical irradiation because of the high activation energy; thus, the photocurrent gradually decays with a long relaxation time, leading to PPC.^31,57^

Lee et al. fabricated an a-IGZO channel-based phototransistor that showed synaptic photoresponses upon irradiation with pulsed ultraviolet (UV) light.^13^ When the UV inputs with low frequency (intensity = 0.6 mW cm^–2^, pulse width = 0.5 s, and frequency = 0.2 Hz) were applied to the a-IGZO phototransistor, the photoconductivity of the a-IGZO phototransistor remained low (Figure 2e). Moreover, when UV inputs with high frequency (frequency = 1.0 Hz) with the same intensity and pulse width were applied to the a-IGZO phototransistor, high photoconductivity was observed (Figure 2f). Such properties are similar to the STP and LTP of synapses, respectively, and are referred to as photon-triggered synaptic plasticity. Furthermore, the a-IGZO phototransistor showed PPC, where its optically programmed conductance gradually relaxed toward the thermodynamically stable initial state upon the removal of the UV inputs. This is similar to the memory effect of synapses.

The synaptic properties can be optimized according to the requirement by introducing a ferroelectric dielectric (e.g., HfZrO_*x*_) into the a-IGZO phototransistor.^58^ HfZrO_*x*_ can be polarized in a downward (or upward) direction by negative (or positive) gate voltages, inducing an additional electric field that inhibits (or facilitates) oxygen vacancy recombination in the a-IGZO channel. The downward polarization causes the depletion of free electrons at the interface between a-IGZO and HfZrO_*x*_, thereby suppressing the recombination reaction (Figure 2g). In contrast, upward polarization causes the accumulation of free electrons at the interface between a-IGZO and HfZrO_*x*_, consequently accelerating the recombination reaction. Moreover, the magnitude of the polarization (e.g., interstates 1 and 2) can be controlled by applying gate voltages of different amplitudes. Therefore, PPC behavior of the a-IGZO phototransistor can be modulated by employing different polarization states (Figure 2h).

Recently, an optoelectronic resistive random-access memory (ORRAM) was proposed to confer permanent PPC characteristics to synaptic optoelectronic devices.^7^ ORRAM was based on a MoO_*x*_ thin film in which the valence state of Mo is changed from Mo^6+^ (semiconducting state) to Mo^5+^ (metallic state) by UV irradiation. When the MoO_*x*_ thin film was exposed to UV light, electrons and holes were generated. The generated holes then react with the water molecules absorbed in the MoO_*x*_, forming a H_*y*_MoO_*x*_ layer that includes Mo^5+^ ions (Figure 2i). Such a transformation results in the transition of ORRAM from a high-resistance state (HRS) to a low-resistance state (LRS). The variation in the resistance is dependent on the UV dose, which is proportional to the light intensity and duration. Furthermore, this transition in ORRAM is irreversible under ambient conditions. Therefore, ORRAM showed a nonvolatile memory effect, and a relatively permanent PPC was observed (Figure 2j). Meanwhile, the ORRAM can be reset by applying a backward bias that extracts protons from the MoO_*x*_ layer to the Pd electrode and thus returns the ORRAM to HRS.

Another advantage of AOSs is their excellent processability, which enables the fabrication of large-scale and highly uniform devices.^51^ High-quality AOSs can be uniformly formed using vacuum-based processes^26,58^ (e.g., sputtering and atomic layer deposition) and/or solution-based processes^28,29^ (e.g., spin coating, inkjet printing, and drop casting). Hong et al. developed an active-matrix synaptic optoelectronic device using a heterostructure of sputtered a-IGZO and solution-processed a-IZO.^17^ The a-IGZO/a-IZO phototransistor exhibited photon-triggered synaptic plasticity and PPC, with excellent pixel-to-pixel uniformity. Therefore, an 8 × 8 active-matrix synaptic optoelectronic device array, in which each pixel consisted of a synaptic phototransistor and a select transistor, could be fabricated (Figures 2k and 2l). Besides, its fabrication is compatible with conventional CMOS fabrication processes, providing considerable potential for integrating synaptic optoelectronic devices on a CMOS chip.

### 2.2 2D Materials for Synaptic Optoelectronic Devices

**Figure 3:**
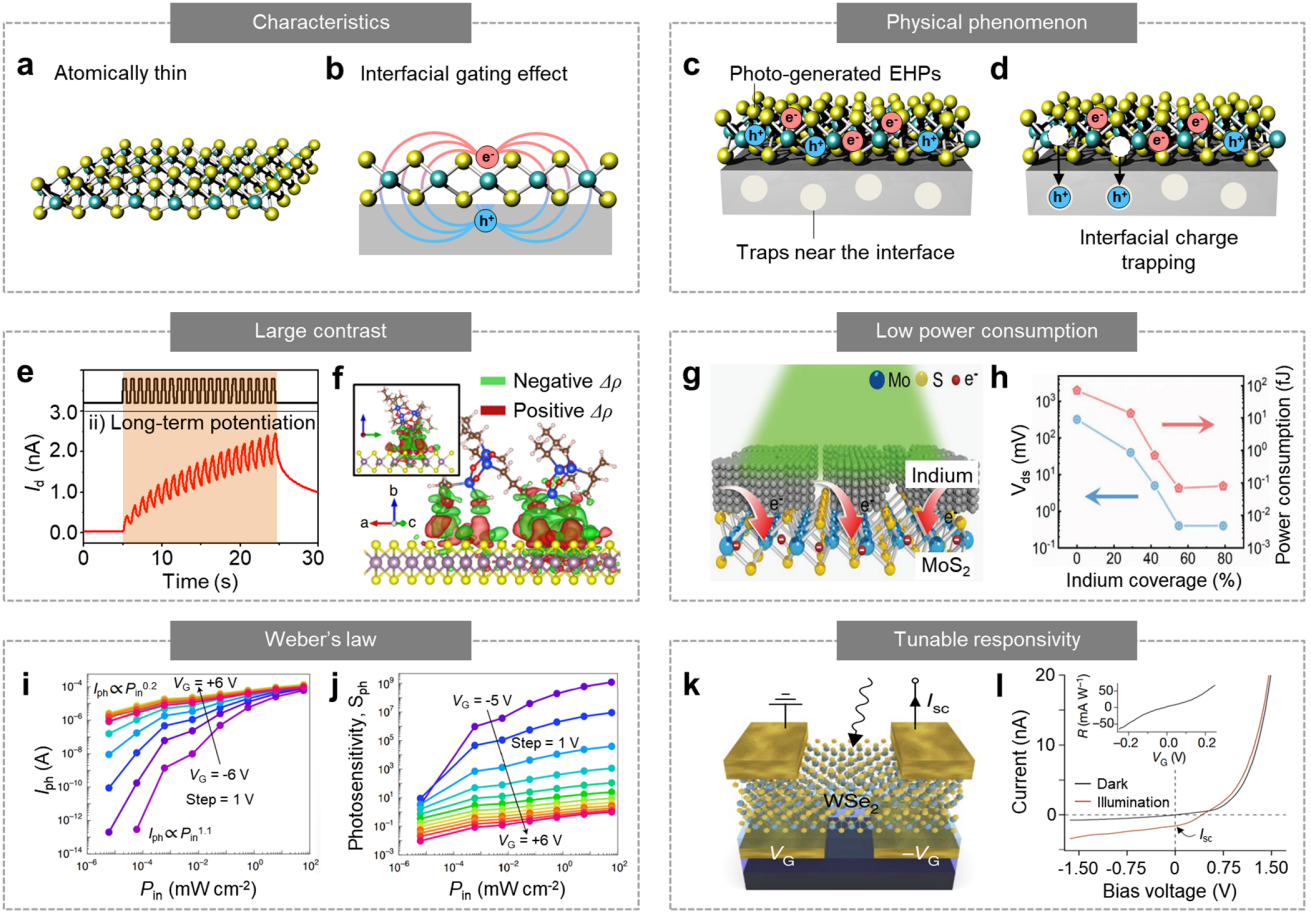
Synaptic optoelectronic devices based on 2D materials. (a) Schematic of the crystal structure of TMDC (e.g., MoS_2_) with atomically thin thickness. (b) Schematic of the electric field generated by an electron and a hole that is trapped in the interface between the 2D material and gate dielectric. (c) Schematic of EHP generation in the 2D materials by optical irradiation. (d) Schematic of interfacial hole trapping that induces a photogating effect. (e) The LTP behavior of the MoS_2_–pV3D3 phototransistor in response to frequent optical pulses (i.e., 20 optical pulses with 0.5 s interval and 0.5 s duration). (f) Charge density difference (Δρ) spatially distributed in the MoS_2_–pV3D3 heterostructure. Green and red contours indicate the potential hole and electron trapping sites, respectively. The inset shows a side view of Figure 3f. Reproduced with permission from ref (21). Copyright 2020 NPG. (g) Schematic of the MoS_2_ surface covered with a discontinuous In layer. The excessive electrons in the In islands are injected into MoS_2_, indicating surface charge transfer doping. (h) Readout voltages and power consumption of the MoS_2_/In phototransistor depending on the In island coverage. Reproduced with permission from ref (75). Copyright 2021 Wiley-VCH. (i, j) Photocurrent (*I*_ph_) (i) and photosensitivity (*S*_ph_) (j) of defect-rich MoS_2_ phototransistors as a function of light intensities (*P*_in_) and the gate bias (*V*_g_). Reproduced with permission from ref (9). Copyright 2022 NPG. (k) Schematic of the WSe_2_ photodiode with split-gate electrodes. (l) *I*–*V* characteristics of the WSe_2_ photodiode under dark and optically irradiated conditions. The inset shows the responsivity of the WSe_2_ photodiode depending on applied *V*_g_. Reproduced with permission from ref (8). Copyright 2020 NPG.

2D materials are atomically thin-layered nanomaterials, and they exhibit unique electrical and optical properties that are not found in their bulk counterparts.^59−61^ It is because their ultrathin thickness introduces a quantum confinement effect (Figure 3a).^62^ Among the 2D materials, transition metal dichalcogenides (TMDCs; e.g., MoS_2_, WS_2_, and WSe_2_) have recently been highlighted for the development of synaptic optoelectronic devices.^63−66^ TMDCs have been used as semiconducting channels in various device configurations, including phototransistors^60,67,68^ and photodiodes.^69,70^ The conductance of ultrathin TMDCs is strongly influenced by the charges trapped at the material or interfacial defects, resulting in synaptic photoresponses (Figure 3b).^71^ For example, TMDC generates electron–hole pairs (EHPs) in response to optical irradiation. The EHPs can spontaneously dissociate, and some of the charges (e.g., holes or electrons) can be trapped at nearby trap sites distributed in the material and adjacent interface (Figure 3c).^20^ Once the charges are trapped at the trap sites, the trapped charges induce an electric field in the TMDC, thus modulating the conductance of TMDC (Figure 3d).^72−74^ This phenomenon is known as the photogating effect. The charge trapping is a slow process because of its large activation energy, leading to time-dependent photocurrent generation. Furthermore, the charges remain partially trapped although the optical irradiation is turned off. It is because the charge detrapping process also requires sufficient energy to overcome the activation energy,^73^ and thereby PPC behavior is observed. However, the conductance can be returned to the initial state by facilitating the detrapping process, for example, by applying a positive bias to a gate electrode of a phototransistor.^75^

For example, Choi et al. developed a synaptic phototransistor based on the heterostructure of MoS_2_ and poly(1,3,5-trimethyl-1,3,5-trivinyl cyclotrisiloxane) (pV3D3).^21^ Its photoresponse resembles STP and LTP of a human synapse, where a high photocurrent is generated by optical inputs with high frequency (i.e., LTP) and a low photocurrent is generated by optical inputs with low frequency (i.e., STP) (Figure 3e). The contrast between these photocurrents could be larger than that of a control device (i.e., conventional MoS_2_-based phototransistor using the Al_2_O_3_ gate dielectric). This is because of the heterostructure of MoS_2_ and pV3D3, in which the charge trap sites are inhomogeneously distributed at the interface due to the irregular geometry of the pV3D3 structure (Figure 3f). Because of the spatially and energetically complex potential hole-trapping sites, the MoS_2_–pV3D3 phototransistor showed quasi-linearly time-dependent photocurrent generation. It differs from the control device, which exhibited nonlinear time-dependent photocurrent generation. Therefore, more photocurrent was generated in the MoS_2_–pV3D3 phototransistor as more optical pulses were applied (Figure 3f).

Another advantage of 2D materials is low power consumption owing to their ultrathin thickness.^44^ Recently, a synaptic optoelectronic device that can achieve ultralow power consumption was developed by depositing discontinuous indium (In) layers onto the MoS_2_ surface.^75^ Because the abundant free electrons in the In islands were injected into MoS_2_ (Figure 3g), the synaptic optoelectronic device could be operated at a lower voltage while maintaining an equivalent magnitude of current density. In particular, surface charge transfer doping was enhanced with an increasing coverage ratio of the In layer. Therefore, the power consumption per spike, which is calculated using the formula *E* = *I* × *V* × *t* (*I*, *V*, and *t* represent the current, applied voltage, and duration of the optical spike, respectively), could be reduced from the femto J level to the atto J level (Figure 3h), significantly improving energy efficiency.

Additionally, 2D materials have been used to emulate Weber’s law, where the sensitivity of the retina is high or low in dim or bright environments, respectively.^76^ To realize this behavior, Liao et al. fabricated a defect-rich MoS_2_ phototransistor using an UV/ozone treatment.^9^ The photoresponse of the defect-rich MoS_2_ phototransistor depends on the applied gate bias (*V*_g_) (Figure 3i). When a negative *V*_g_ is applied, the photocurrent generation is almost proportional to the light intensity (*P*_in_) because the photoconductive effect is dominant. However, when a positive *V*_g_ is applied, the photocurrent generation is sublinearly proportional to *P*_in_ because the photogating effect becomes dominant.^77^ The defect-rich nature of the phototransistor enhances the photogating effect. Therefore, photosensitivity (*S*_ph_), which is defined as the ratio of the photocurrent (*I*_ph_) to the dark current (*I*_dark_), can be tuned by modulating *V*_g_. This relationship between *S*_ph_ and *V*_g_ is similar to Weber’s law, where the sensitivity of the retina is dependent on the background light intensity (Figure 3j).

Using the electrostatically tunable photoresponse of 2D materials, a WSe_2_ photodiode with tunable responsivity was developed.^8^ The WSe_2_ photodiode had split-gate electrodes, each of which was biased with opposite voltages (*V*_g_ and −*V*_g_) (Figure 3k). Such split-gate biasing induces electrostatic doping in two different regions in the WSe_2_ channel, forming a lateral p–n photodiode. In addition, the responsivity of the WSe_2_ photodiode could be modulated from −60 mA W^–1^ to 60 mA W^–1^ by changing the magnitude of *V*_g_ (Figure 3l). This enables ambipolar conduction behavior, in which the responsivity can be tuned to the desired value, corresponding to the synaptic weight of an artificial neural network (ANN).

### 2.3 Semiconducting Nanoparticles for Synaptic Optoelectronic Devices

**Figure 4:**
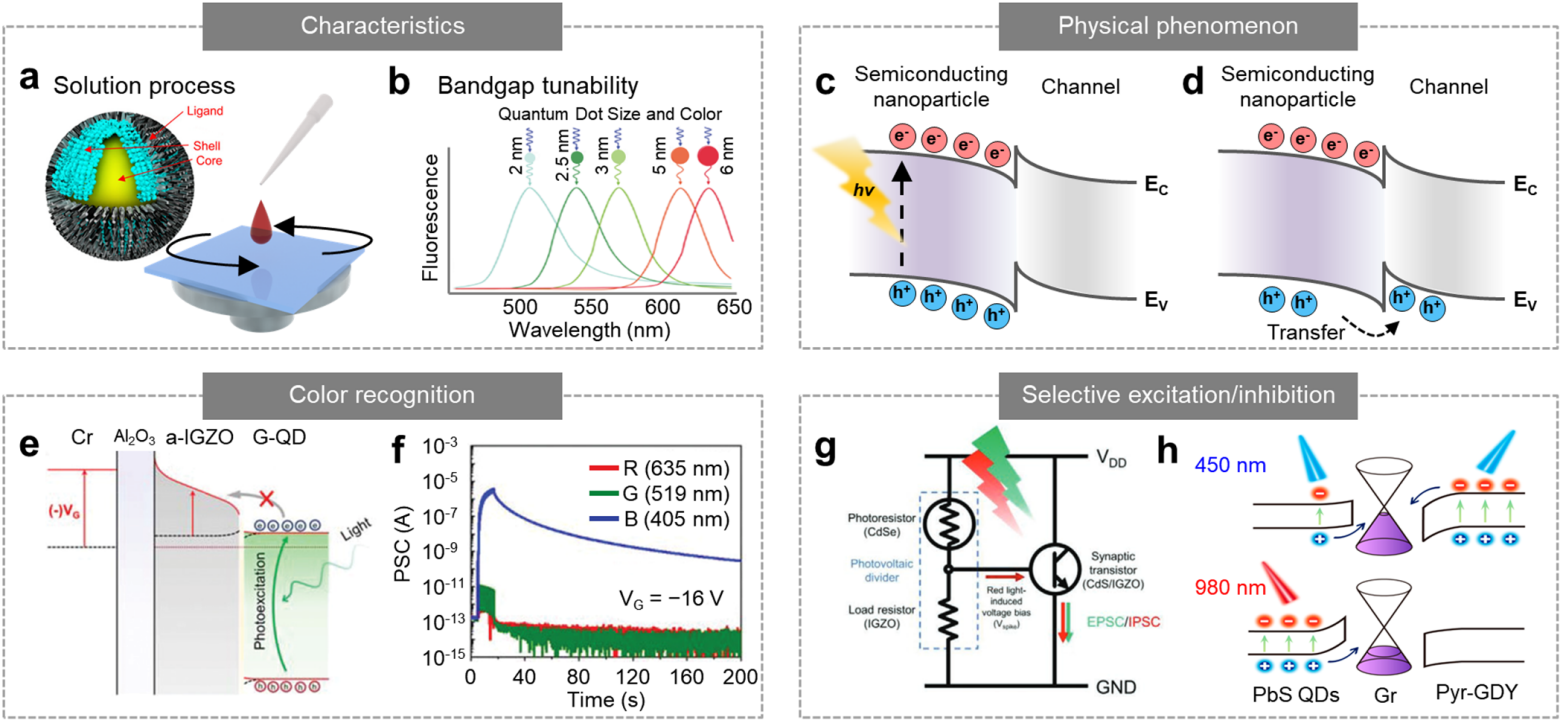
Synaptic optoelectronic devices based on semiconducting nanoparticles. (a) Schematic of the fabrication process of the optoelectronic device based on the semiconducting nanoparticle solution. (b) Photoluminescence spectra of QDs of different sizes. The bandgap of QDs is dependent on their sizes. (c,d) Energy band diagram of the heterointerface between semiconducting nanoparticles and adjacent channel material under light irradiation. The EHPs are generated by the optical inputs (c), and the charges are dissociated at the interface (d). (e) Energy band diagram of the heterointerface between green QDs and a-IGZO under green light illumination. The gate bias of −16 V was applied to the device. (f) Photocurrent generation and decay characteristics of the color-recognitive synaptic phototransistor under the irradiation of red, blue, and green light. The gate bias was set to −16 V. Reproduced with permission from ref (94). Copyright 2022 Wiley-VCH. (g) Circuit design of a fully optically triggered artificial synapse composed of a CdS/a-IGZO synaptic phototransistor and voltage divider of a CdSe photoresistor and a-IGZO load resistor. Reproduced with permission from ref (10). Copyright 2021 Wiley-VCH. (h) Energy band diagram of a PbS QD/graphene/Pyr-GDY heterostructure under the irradiation of a 450 nm (top) and 980 nm (bottom) laser. Reproduced with permission from ref (98). Copyright 2021 ACS.

Semiconducting nanoparticles have been widely used in optoelectronic devices (e.g., image sensors^78,79^ and displays^80−82^) owing to their facile processability and excellent optical characteristics (e.g., high color purity and high quantum efficiency).^83,84^ Semiconducting nanoparticles can be coated as a thin film via low-temperature solution-based processes (Figure 4a), allowing integration with other functional nanomaterials (e.g., AOSs and 2D materials).^85,86^ These semiconducting nanomaterials have diverse form factors ranging from 0D (i.e., quantum dots (QD)) to 3D bulk. Among these, QDs have attracted significant attention because of their size-dependent characteristics arising from the quantum confinement effect.^83,85^ For example, the bandgap of QDs can be precisely tuned by engineering the degree of confinement within the range of exciton Bohr radii (2–20 nm).^87^ Therefore, QDs with different diameters exhibit size-dependent optical and electrical properties, such as photoluminescence spectra and conduction band minima (*E*_c_) (Figure 4b).^88,89^

Type-II heterostructures of semiconducting nanoparticles and adjacent functional nanomaterials have also been used in synaptic optoelectronic devices because slow charge transfer at their heterojunction leads to synaptic photoresponses.^90,91^ A large amount of EHPs are generated in the semiconducting nanoparticles when irradiated with light (Figure 4c).^77,78^ Then, the generated electrons and holes drift in opposite directions owing to the band offset formed in the type-II heterostructure.^92^ Electrons and holes accumulate at each material across the interface, increasing the photoconductivity (Figure 4d).^30,92^ Such charge transfer is energetically favorable for type-II band alignment but still requires high activation energy. Therefore, heterostructures composed of semiconducting nanoparticles show unconventional photoresponses (e.g., time-dependent photocurrent generation and PPC).

Inspired by the human retina, which allows for multispectral color perception,^93^ Jo et al. reported a color-cognitive synaptic phototransistor by monolithically integrating a mixed QD layer with an a-IGZO thin film.^94^ The QD layer was composed of a mixture of red, green, and blue QDs with diameters of approximately 7.1, 4.4, and 3.3 nm, respectively. Such QDs were selectively excited depending on the wavelength of the incident light owing to their different bandgaps.^87^ Considering the different responsivities of QDs, the mass ratio of red, green, and blue QDs was precisely designed to be 0.5:1.0:8.5 for effective color discrimination. The synaptic phototransistor can selectively detect colored light by modulating the conduction barrier of the a-IGZO. Because the negative gate bias increases the *E*_c_ of the a-IGZO channel, the conduction barrier becomes larger as a larger negative gate bias is applied, and consequently, electron transfer from the QDs to the a-IGZO channel is hindered (Figure 4e). For QDs with larger *E*_c_, a larger negative bias is required to sufficiently increase the barrier for inhibiting charge transfer. For example, gate biases of −8 V for the red QDs and −16 V for the green QDs are required to inhibit charge transfer because *E*_c_ is larger in the order of blue, green, and red QDs. Therefore, when a gate bias of −16 V was applied, the photocurrent generated by the red and green light was noticeably suppressed below 10^–11^ A, whereas the photocurrent generated by the blue light was over 10^–6^ A, indicating color recognition was performed by the single device component (Figure 4f).

The human synapses show both excitatory and inhibitory responses by the presynaptic action potentials.^95^ Inspired by these responses, electrical synaptic devices exhibiting both excitatory and inhibitory behaviors have been used for neuromorphic computing, providing the advantages of ultrafast computational speed with low crosstalk, high bandwidth, and low power consumption.^49^ Recently, optically triggered artificial synapses that exhibit both excitatory and inhibitory behaviors have been developed by integrating semiconducting nanoparticles with different bandgaps.^95−97^ The artificial synapse is composed of a CdS/a-IGZO synaptic phototransistor, in which the gate electrode is connected to a voltage divider comprising a CdSe photoresistor and an a-IGZO load resistor (Figure 4g).^10^ Because the bandgap of CdS and a-IGZO is 2.36 and 3.69 eV, respectively, the CdS/a-IGZO synaptic phototransistor generated the photocurrent in response to the green light because of the charge transfer between the CdS and a-IGZO, which corresponds to excitatory behavior. In contrast, the CdS/a-IGZO synaptic phototransistor generated no photocurrent upon irradiation with red light because of its large bandgap. Meanwhile, irradiation with red light reduced the resistance of the CdSe photoresistor, whose bandgap is ∼1.7 eV. Subsequently, the voltage applied to the gate electrode of the synaptic phototransistor increased, and a large gate bias induced the detrapping of electrons accumulated in the a-IGZO channel. Thus, the photoconductivity decreased, which corresponds to the inhibitory behavior.

Light-mediated excitation and inhibition have also been realized at the single-device level using the PbS QD/graphene/pyrenyl graphdiyne (Pyr-GDY) heterostructure.^98^ Upward and downward band bending could be achieved at the PbS QD/graphene interface and the Pyr-GDY/graphene interface, respectively, because of the work function mismatch between these materials. When 450 nm light is irradiated, it is strongly absorbed by the topmost Pyr-GDY more than the bottom PbS QDs, generating EHPs mostly in the Pyr-GDY film (Figure 4h, top). The built-in electrical field then promotes electron transfer from Pyr-GDY to graphene, and the holes are trapped at Pyr-GDY and vice versa for PbS QDs. However, because the number of trapped holes in Pyr-GDY is considerably larger than that of trapped electrons in PbS QDs, the conductivity of graphene, a hole-dominated channel,^71^ is reduced (i.e., positive photogating effect). Conversely, when the device was irradiated with 980 nm light, the EHPs were only generated in the PbS QDs (Figure 4h, bottom). Therefore, electron trapping in PbS QDs induces a negative photogating effect, increasing the conductivity of graphene.

### 2.4 Halide Perovskite for Synaptic Optoelectronic Devices

**Figure 5:**
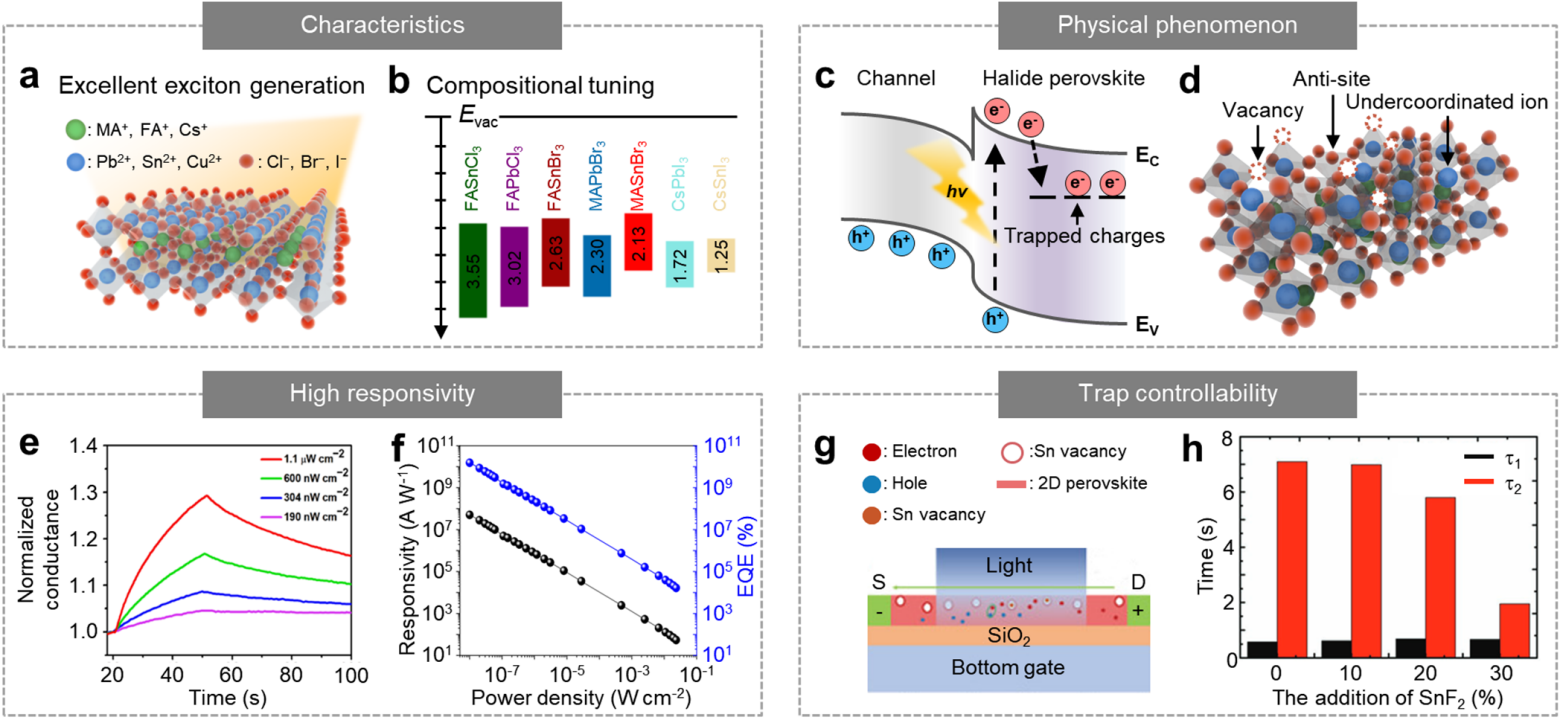
Synaptic optoelectronic devices based on halide perovskites. (a) Schematic of the optical advantages of halide perovskites, such as large photoabsorption and high exciton generation efficiency. (b) Energy band diagram of the diverse halide perovskites with different compositions. (c) Energy band diagram of the heterointerface between the halide perovskite and adjacent channel material. The photogenerated charges in the halide perovskite are transferred to the channel, and the remaining charges are trapped in the defects, inducing a photogating effect. (d) Schematic of various kinds of defects in the halide perovskites. (e) Photocurrent generation and decay characteristics of the synaptic phototransistor based on the CH_3_NH_3_PbBr_3_ QD/graphene heterostructure under the optical irradiation of different intensities. Reproduced with permission from ref (120). Copyright 2020 AAAS. (f) EQE and responsivity of the synaptic optoelectronic device based on the CsPbBr_3_ QD/CNT heterostructure. Reproduced with permission from ref (114). Copyright 2021 NPG. (g) Schematic of a (PEA)_2_SnI_4_-based synaptic photoconductor. The charge trapping is induced by Sn vacancies in the (PEA)_2_SnI_4_. (h) Detrapping time constant for the shallower traps (τ_1_) and the deeper traps (τ_2_) dependent on the amount of Sn vacancy. Reproduced with permission from ref (47). Copyright 2019 Wiley-VCH.

Halide perovskites have been the most highlighted nanomaterials for developing optoelectronic devices because of their high photoabsorption coefficient,^99^ excellent exciton generation efficiency,^100,101^ long carrier lifetime,^102^ and long diffusion length (Figure 5a).^103,104^ Furthermore, the halide perovskite provides tunability of the optical and electrical characteristics through the modulation of the composition.^105^ It has the general formula ABX_3_, where A is a large cation (e.g., MA^+^ = CH_3_NH_3_^+^, FA^+^ = CH_3_ (NH_2_)_2_^+^, and Cs^+^); B is a divalent transition metal (e.g., Pb^2+^, Sn^2+^, and Cu^2+^); and X is a halide anion (e.g., Cl^–^, Br^–^, and I^–^).^106^ Various halide perovskite material candidates can be synthesized through a combination of such components, each of which exhibits distinguishable characteristics (e.g., energy band structure) (Figure 5b).^107^

Halide perovskites have considerable potential for high-performance synaptic optoelectronic devices.^108−112^ Halide perovskites strongly absorb light and generate EHPs with high efficiency.^113^ The photogenerated EHPs can be effectively dissociated at the interface between the halide perovskite and adjacent channel material owing to the built-in electric field. The dissociated holes can then be transferred to the channel material, whereas the remaining electrons can be trapped in the intrinsic defects of the halide perovskite (Figure 5c).^114,115^ There are various types of intrinsic defects in halide perovskites, such as vacancies, antisites, and undercoordinated ions.^104^ Such defects may become potential charge trap sites that induce a photogating effect through capacitive coupling (Figure 5d).^116^ Because charge transfer at the heterointerface and internal charge trapping require high activation energy, synaptic photoresponses can be observed in the perovskite-based optoelectronic devices.^117−119^

Recently, a high-performance synaptic optoelectronic device was developed using a heterostructure of perovskite QDs (e.g., CH_3_NH_3_PbBr_3_) and graphene.^114,120^ Although graphene shows high carrier mobility exceeding that of conventional semiconducting materials, its weak photoabsorption and low exciton generation efficiency make it challenging to fabricate a high-performance optoelectronic device. In this regard, Pradhan et al. developed synaptic phototransistors exhibiting exciton generation efficiency and excellent charge transport characteristics as well as synaptic properties by integrating perovskite QDs with graphene (Figure 5e).^120^ Perovskite QDs generate a significant amount of EHPs in response to light irradiation, and the generated electrons and holes are separated at the interface between the perovskite QDs and graphene because of the built-in electric field. Then, the excess electrons are trapped at the intrinsic defects of the perovskite QDs, which induces a photogating effect and significantly enhances the conductivity of graphene. Therefore, this device exhibited a responsivity of 1.4 × 10^8^ A W^–1^ and a specific detectivity of 4.72 × 10^15^ Jones. Using a similar device strategy, Zhu et al. developed a synaptic optoelectronic device based on a heterostructure of perovskite QDs (e.g., CsPbBr_3_) and carbon nanotubes.^114^ This device achieved high performance with an external quantum efficiency (EQE) of 1.6 × 10^10^%, a responsivity of 5.1 × 10^7^ A W^–1^, and a specific detectivity of 2 × 10^16^ Jones (Figure 5f).

Because the optical and electrical characteristics of halide perovskites can be engineered by adjusting their composition, a synaptic photoconductor with tunable photoelectrical properties could be developed. It is based on a 2D layered perovskite (e.g., (PEA)_2_SnI_4_), which exhibits paired-pulse facilitation, short-term memory, and long-term memory (Figure 5g).^47^ Such synaptic properties arise from photogenerated electrons trapped in positive Sn vacancies. The trapped electrons generate a photocurrent owing to the photogating effect and also cause retention properties because of the long detrapping time. In addition, by controlling the amount of Sn vacancies by adding SnF_2_, the trapping/detrapping process can be modulated. That is, the detrapping time constant for deeper traps decreases as Sn vacancies are suppressed (Figure 5h). Furthermore, a blue shift in the absorption spectrum of (PEA)_2_SnI_4_ can be achieved by partially replacing I^–^ with Br^–^, providing wavelength tunability to the synaptic optoelectronic device.

## 3 In-Sensor Preprocessing by Synaptic Optoelectronic Devices

Conventional imaging and data processing systems use frame-based image data acquisition and processing.^35,121,122^ The image data of the individual timeframes are captured by the image sensor.^20^ Then, a significant amount of raw data obtained over the entire time domain are stored in a memory device and transferred to a processor for image recognition.^123^ Image preprocessing, which extracts important features from the images, can facilitate postprocessing, such as image recognition.^124^ However, handling a large amount of raw data requires high power consumption and long processing time. Therefore, this front-end part of machine vision can be inefficient.^125^ In contrast, synaptic optoelectronic devices perform image preprocessing at the image-sensor level.^16^ Through in-sensor preprocessing performed by the image-sensing device itself, the preprocessed data can be obtained from massive raw image data without computationally expensive preprocessing steps.^5^ In the following sections, we will describe advantages of the in-sensor preprocessing in more detail.

### 3.1 Contrast Enhancement by Synaptic Optoelectronic Devices

**Figure 6:**
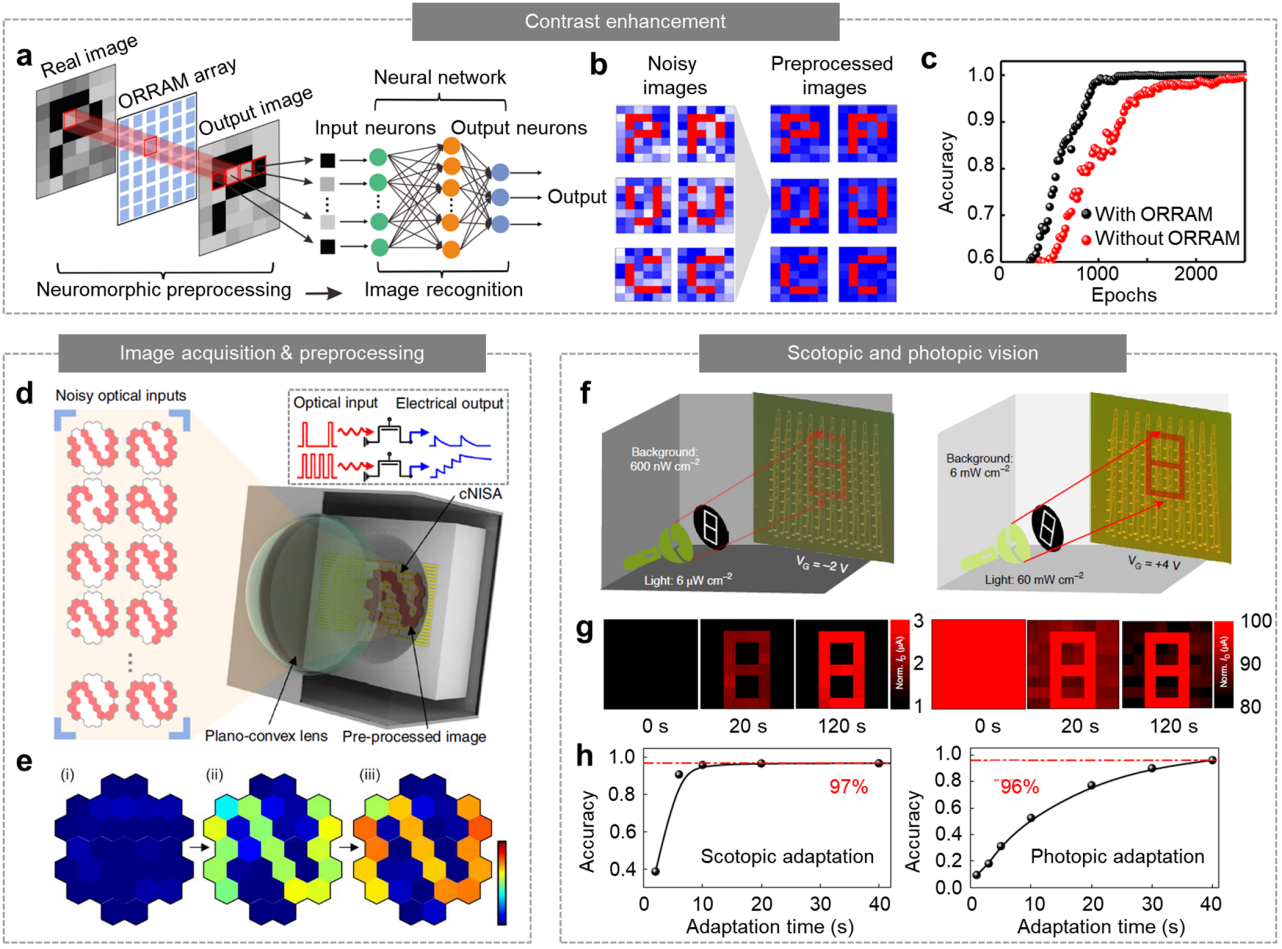
Contrast enhancement by synaptic optoelectronic devices. (a) Schematic of contrast enhancement using ORRAM and image recognition using an ANN. (b) In-sensor preprocessed images with enhanced contrast compared with the noisy raw images. (c) Recognition accuracy of the preprocessed images and noisy raw images. Reproduced with permission from ref (7). Copyright 2019 NPG. (d) Schematic of a curved synaptic image sensor array inspired by the human retina. The inset shows the photon-triggered synaptic plasticity of the MoS_2_–pV3D3 phototransistor. (e) Patterns acquired using the curved synaptic image sensor array through real-time image acquisition and in-sensor preprocessing. Reproduced with permission from ref (21). Copyright 2020 NPG. (f) Schematic showing the concept of in-sensor visual adaptation. (g) Preprocessed images acquired by the defect-rich MoS_2_ phototransistor array through in-sensor visual adaptation for scotopic vision (left) and photopic vision (right). (h) Recognition accuracy of in-sensor preprocessed images via scotopic and photopic adaptation over the adaptation time. Reproduced with permission from ref (9). Copyright 2022 NPG.

For image recognition, the image data captured by the imaging module were classified using a pretrained ANN (Figure 6a, right).^7^ The intensity of each image pixel was applied to the input nodes and multiplied by the synaptic weights while propagating the hidden layers. The values of the output nodes that indicate Bayesian probabilities were then compared to classify the image. During this process, background noise in the image data can decrease the image recognition rate.^15^ Zhou et al. demonstrated contrast enhancement of image data through in-sensor preprocessing by using ORRAM (Figure 6a, left).^7^ The ORRAM generated a weighted photocurrent in proportion to the light dosage (e.g., irradiation time and intensity) and showed PPC with a long retention time (Figure 2j). Therefore, the contrast of the output images increased as the noisy image was illuminated to the ORRAM longer, and thus the contrast-enhanced images could be acquired (Figure 6b). Compared with the background noise, strong signals were highlighted. The preprocessed images could be recognized using an ANN with higher accuracy compared with raw noisy images (Figure 6c), proving the potential of in-sensor preprocessing for machine vision.

Bioinspired concepts have also been adopted to simplify optical systems and improve image data preprocessing. The human eye can achieve aberration-free imaging using a single lens because the hemispherical retina matches the hemispherical focal plane formed by the lens.^20^ Inspired by the hemispherical retina, a curved form of a synaptic image sensor array was developed (Figure 6d).^21^ This device is deformable because of the intrinsically flexible nanomaterials (e.g., MoS_2_, graphene, and pV3D3), ultralow device thickness (∼75 nm), and mesh-shaped device design. Therefore, it can be fabricated on the hemispherical substrate. In addition, the phototransistor based on the MoS_2_–pV3D3 heterostructure exhibited photon-triggered synaptic plasticity, producing either a highly weighted and long-lasting photocurrent by optical inputs with high frequency or a low-level and quickly decaying photocurrent by optical inputs with low frequency (i.e., LTP and STP, respectively; Figure 6d inset). Therefore, the curved synaptic image sensor array can derive a preprocessed image from the sequentially irradiated noisy optical inputs, by which the contrast is enhanced and the noise is reduced (Figures 6d and 6e). The miniaturization of the optical system was also enabled because of the curved synaptic image sensor array that matched with a curved focal plane formed by a single planoconvex lens.

The synaptic optoelectronic device mimicking Weber’s law was also used for scotopic and photopic vision (Figure 6f).^126^ The human eye can effectively detect objects in both dim and bright environments using a visual adaptation function.^76^ Similarly, the defect-rich MoS_2_ phototransistor array, whose photosensitivity can be tuned depending on the background light intensity by modulating the gate bias (Figure 3j), can successfully capture target images under a dim or bright environment, respectively. Under a dim environment, a negative gate bias is applied, which induces electron detrapping at the MoS_2_ trap sites. Therefore, the photocurrent generated by the weak signals is accumulated, whereas the photocurrent generated by the dim background is negligibly increased. In contrast, under a bright environment, a positive gate bias is applied, which induces electron trapping at the MoS_2_ trap sites. Therefore, the photocurrent generated by the bright background is decreased, whereas the photocurrent generated by the strong signals is barely decreased. As a result, the preprocessed outputs, which were not discernible at the early stage of imaging, became clearer over the adaptation time (Figure 6g). Such preprocessed images can be recognized using an ANN with high accuracy as the adaptation time increases, and the accuracy reaches over 96% after tens of seconds (Figure 6h).

### 3.2 Image Filtering and Pattern Classification by Synaptic Optoelectronic Devices

**Figure 7:**
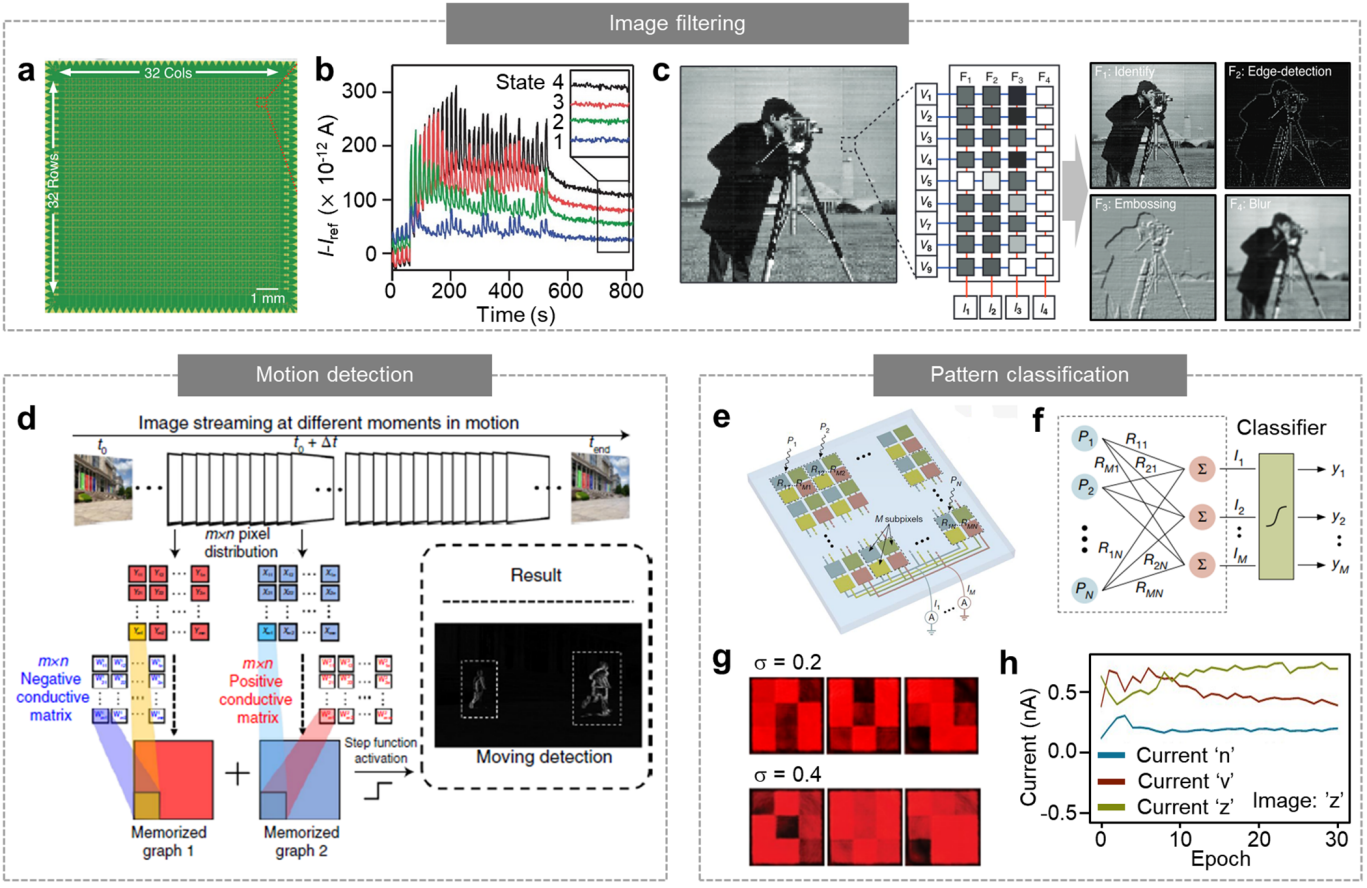
Image filtering and pattern classification by synaptic optoelectronic devices. (a) 32 × 32 MoS_2_ phototransistor array for image acquisition and analog vector-matrix multiplication. (b) Iterative optical programming of MoS_2_ phototransistors to obtain four different conductance states. (c) Image filtering using a MoS_2_ phototransistor array. The intensity of each 3 × 3 pixel in the cameraman image is applied to the conductance matrices programmed for each filter (middle), resulting in the filtered images (right). Reproduced with permission from ref (14). Copyright 2020 Wiley-VCH. (d) Schematic of moving object detection by interframe differencing computations. Reproduced with permission from ref (129). Copyright 2022 NPG. (e) Schematic of the photodiode array constituting an ANN. (f) Schematic of the in-sensor pattern classification. (g) Patterns with a noise level of 0.2 (top) and 0.4 (bottom). Those patterns were illuminated on the photodiode array. (h) Output currents from each subpixel at different training epochs. The output currents indicate the Bayesian probability of each letter. Reproduced with permission from ref (8). Copyright 2020 NPG.

The roles of synaptic optoelectronic devices have been expanding to image filtering^5,14,127^ and pattern classification,^8,16^ which were originally achieved using conventional microprocessors with appropriate software. Synaptic optoelectronic devices can serve as image filters and ANNs, which can be used for feature extraction and image recognition.^127,128^ For example, Jang et al. reported a 32 × 32 MoS_2_ phototransistor array (Figure 7a), which captures incoming images as image sensors and also functions as a matrix multiplication engine for image filtering and recognition.^14^ Owing to the PPC of the MoS_2_ phototransistor, the conductance of each pixel can be modulated via optical encoding (Figure 7b). Therefore, the conductance matrix can be set to the desired values corresponding to the 9 × 1 vector converted from the 3 × 3 kernels for image filtering (e.g., identify, edge detection, embossing, and blur), and filtering of the acquired images was achieved via analog vector-matrix multiplication (Figure 7c). The phototransistor array was also used for recognizing handwritten digits.^14^ By programming the conductance matrix to represent the convolutional layers of the convolutional neural network, an input image was classified by comparing the outputs that presented the Bayesian probabilities.

Image filtering for detecting moving objects was also demonstrated using a synaptic optoelectronic device based on a BP/Al_2_O_3_/WSe_2_/h-BN heterostructure.^129^ The floating-gate structure of this device was initially programmed using electrical pulses (that is, electrons or holes were accumulated in WSe_2_), and the conductance increased or decreased in response to the incident light depending on the initial programming, leading to positive or negative photoconductivity, respectively. Owing to these attributes, the positive and negative photoconductivity matrices could be prepared. Then, the detected images at *t*_0_ and *t*_0_ + Δ*t* were multiplied with positive and negative photoconductivity matrices, respectively, and they were summed to extract the moving objects while erasing the unchanging ones (Figure 7d). The acquired image of the moving objects can be recognized using an ANN with high accuracy within a small number of epochs, although the noise added to the original images might hinder the recognition.

The hardware-based classification of the incident patterns was also realized.^8^ Mennel et al. reported the WSe_2_ photodiode array that performs pattern classification by itself, where the responsivity of each pixel was tuned as the synaptic weight of the ANN (Figure 3l). The array had *N* pixels, and each comprised *M* subpixels (Figure 7e). Each subpixel consisted of a WSe_2_ photodiode with tunable responsivity. Therefore, when the optical input consisting of *N* pixels (e.g., P_1_, P_2_,···, and P_*N*_) was irradiated to the photodiode array, each pixel generated *M* current outputs in their subpixels, and the outputs of the *m*^th^ subpixels were summed (*I*_*m*_ = Σ*R*_*mn*_*P*_*n*_) (Figure 7f). Each output (*I*_*m*_) indicated the Bayesian probabilities used for pattern classification. As a demonstration, the 3 × 3 × 3 photodiode array successfully classified the pattern of “*n*”, “*v*”, and “*z*” with a high noise level (Figure 7g). The responsivity of each photodiode was trained through backpropagation; thus, the incident image could be identified by comparing the current outputs from each subpixel (Figure 7h).

## 4 Conclusions and prospects

In this review, we discuss recent progress in the development of synaptic optoelectronic devices using functional nanomaterials and their advantageous applications to in-sensor preprocessing for achieving efficient machine vision. Owing to the unique physics of functional nanomaterials (e.g., oxygen vacancy ionization of AOSs, interfacial charge trapping of 2D materials, and heterojunction charge transfer of semiconducting nanoparticles and halide perovskites), synaptic optoelectronic devices based on such nanomaterials have shown photon-triggered synaptic plasticity (i.e., STP and LTP triggered by optical stimuli) and PPC. Moreover, functional nanomaterials have enabled unique properties such as permanent PPC, tunable responsivity, color selectivity, and ultrahigh responsivity, all of which are beneficial for advancing synaptic optoelectronic devices. With such attributes, synaptic optoelectronic devices have been used to perform in-sensor preprocessing, such as contrast enhancement and image filtering, while performing their original role as image sensors. Therefore, synaptic optoelectronic devices offer a promising image sensing and processing paradigm for next-generation machine-vision systems.

However, various challenges still remain in developing high-performance synaptic optoelectronic devices using functional nanomaterials. First, it is necessary to consider the inherent strengths and weaknesses of materials in consideration of the target application. For example, AOSs can be monolithically integrated with conventional CMOS systems. However, because AOSs usually have a large bandgap, their application to image acquisition and preprocessing of visible and infrared information is limited. Despite the quantum confinement effect and low power consumption of 2D materials, their integration level is not sufficient compared to that of conventional CMOS technology, which needs further breakthroughs for achieving large-scale, high-density, and uniform integration of 2D material-based synaptic optoelectronic devices. For semiconducting nanoparticles and halide perovskites, it is necessary to improve high-resolution patterning strategies and encapsulation techniques that could improve the processability and air stability of synaptic optoelectronic devices.^130^

In addition, there is room for innovation in terms of device design and fabrication techniques. There are numerous properties of synapses and neurons that could help achieve image-based applications but have not yet been completely explored. For example, the postsynaptic potential is accumulated by repetitive presynaptic potentials, and the neurons fire the action potential once the postsynaptic potential exceeds the threshold. Such properties can be realized by using external electronic circuits (e.g., comparators and reset circuits) or by integrating two or more optoelectronic components;^131^ however, they are not ideal in terms of power consumption and hardware complexity.^132^ In this regard, the realization of such properties at the single-device level would be beneficial for energy-efficient machine vision as well as module miniaturization. Furthermore, the synaptic optoelectronic device with a nanoscale dimension, comparable to CMOS processors, will significantly improve computing power as well as bandwidth. However, it is difficult to scale down the device dimension due to the diffraction limit of light. In this regard, the monolithic integration of synaptic optoelectronic devices with CMOS processors can be a solution to achieve both efficient preprocessing by the synaptic optoelectronic devices and high-speed digital processing by the CMOS processors, which has been recently highlighted as near-sensor processing of visual information. For such a goal, the nonuniformity issue of synaptic optoelectronic devices should also be addressed.

The synaptic optoelectronic devices have the potential to lead to advances in other fields beyond vision sensing and preprocessing.^133^ For example, these devices can be used in an artificial sensory system inspired by a nociceptor.^134^ The nociceptor responds to an external stimulus beyond a certain intensity, thus effectively and efficiently detecting noxious damages (e.g., mechanical pressure and thermal damage) that can potentially induce serious injury. It can be emulated by using the synaptic properties;^135^ therefore, the synaptic optoelectronic devices can be used in an alerting system to avoid potential damages.

Nevertheless, the synaptic optoelectronic devices are still in their early stages compared to the CMOS technologies. The pixel density and frame rate of synaptic optoelectronic devices are far less than those of conventional CMOS image sensors, resulting in low-resolution image acquisition. Furthermore, in-sensor preprocessing of synaptic optoelectronic devices may cause loss of image information that can be used for specific machine vision applications. The analog image preprocessing performed by the synaptic optoelectronic devices includes errors, which cannot be found in the digital backend of the CMOS processor. Nevertheless, the synaptic optoelectronic devices have a high potential to perform efficient image-based applications in terms of power consumption and data latency and thus can pave the way for a promising image sensing and processing paradigm for the next-generation machine vision system. The exploitation of functional nanomaterials that provide unconventional physical properties would help this goal.
